# Vitamin C Deficiency in an Adolescent With Severe Anorexia Nervosa

**DOI:** 10.7759/cureus.76674

**Published:** 2024-12-31

**Authors:** Brielle Cram, Katherine Pohoreski, Chris Novak, Ellie Vyver

**Affiliations:** 1 Department of Pediatrics, University of Calgary, Calgary, CAN

**Keywords:** adolescent medicine, anorexia nervosa, malnutrition, nutritional deficiencies, scurvy, vitamin c deficiency

## Abstract

This article reports on the case of a 14-year-old non-binary patient with anorexia nervosa - restrictive subtype (AN-R), presenting with severe malnutrition and the cutaneous manifestations of scurvy. Scurvy is an uncommon nutritional deficiency caused by a lack of dietary vitamin C. In this case, we highlight the clinical features of vitamin C deficiency, a rare but treatable nutritional deficiency that should not be overlooked in patients presenting with malnutrition, including those with eating disorders. This case reviews the pathophysiology, common clinical manifestations, and prevention of vitamin C deficiency and highlights pediatric populations at risk for the development of this condition.

## Introduction

Eating disorders are complex biopsychosocial conditions that often begin during adolescence. Anorexia nervosa (AN) is a condition characterized by restriction of dietary intake leading to weight loss and maintenance of severely low body weight, accompanied by a fear of weight gain or a persistent lack of recognition of the seriousness of low body weight [1]. AN is associated with significant morbidity [1] and can lead to severe multisystem medical complications, which may include dermatologic findings such as lanugo hair, alopecia, acrocyanosis, easy bruising, carotenoderma, and petechiae [2]. Patients with AN are also at risk of various micronutrient deficiencies [3], making close attention to history and dermatologic examination paramount.

On an international level, individuals with eating disorders have experienced an increase in symptomatology during the COVID-19 pandemic, reflected by an increase in hospital admissions [4,5]. The exacerbation of eating disorder symptoms is thought to be secondary to decreased access to care, changes to routine, social isolation, and influence of the media [4,5]. In this case report, we promote awareness of a possible nutritional deficiency associated with severe malnutrition due to AN: vitamin C deficiency also referred to as scurvy.

Scurvy, a condition notable for gingival, dermatological, and skeletal changes, results from a prolonged dietary deficiency of vitamin C [6]. While scurvy is rare in the pediatric population, those with unusual dietary habits, mental illness, or physical disabilities are more prone to the condition [6]. Although this nutritional deficiency is not commonly seen in patients living with eating disorders, vitamin C deficiency presents with distinct cutaneous manifestations and is readily treatable with the administration of oral ascorbic acid [7]. This case study presents a 14-year-old patient with a 32 kg weight loss secondary to AN, hyperpigmented perifollicular petechiae on a dermatologic exam, and resolution of dermatologic findings with vitamin C replacement, with a clinical diagnosis of scurvy.

## Case presentation

A previously healthy 14-year-old non-binary youth (assigned female at birth, she/they pronouns) presented to the Emergency Department with a seven-month history of progressive food restriction and a 32 kg weight loss over the same period. Prior to the onset of illness, she had normal growth and development and had achieved normal menarche. The patient estimated her intake at 100 kcal/day over the past month. She disclosed excessive exercise, with no report of purging via self-induced vomiting or laxative/diuretic/diet pill use. Symptoms reported included syncope, cold intolerance, weakness, constipation, and secondary amenorrhea for six months. The patient did not endorse symptoms of easy bleeding, dental concerns, or musculoskeletal pain. No mental health comorbidities were identified at the time of admission.

**Figure 1:**
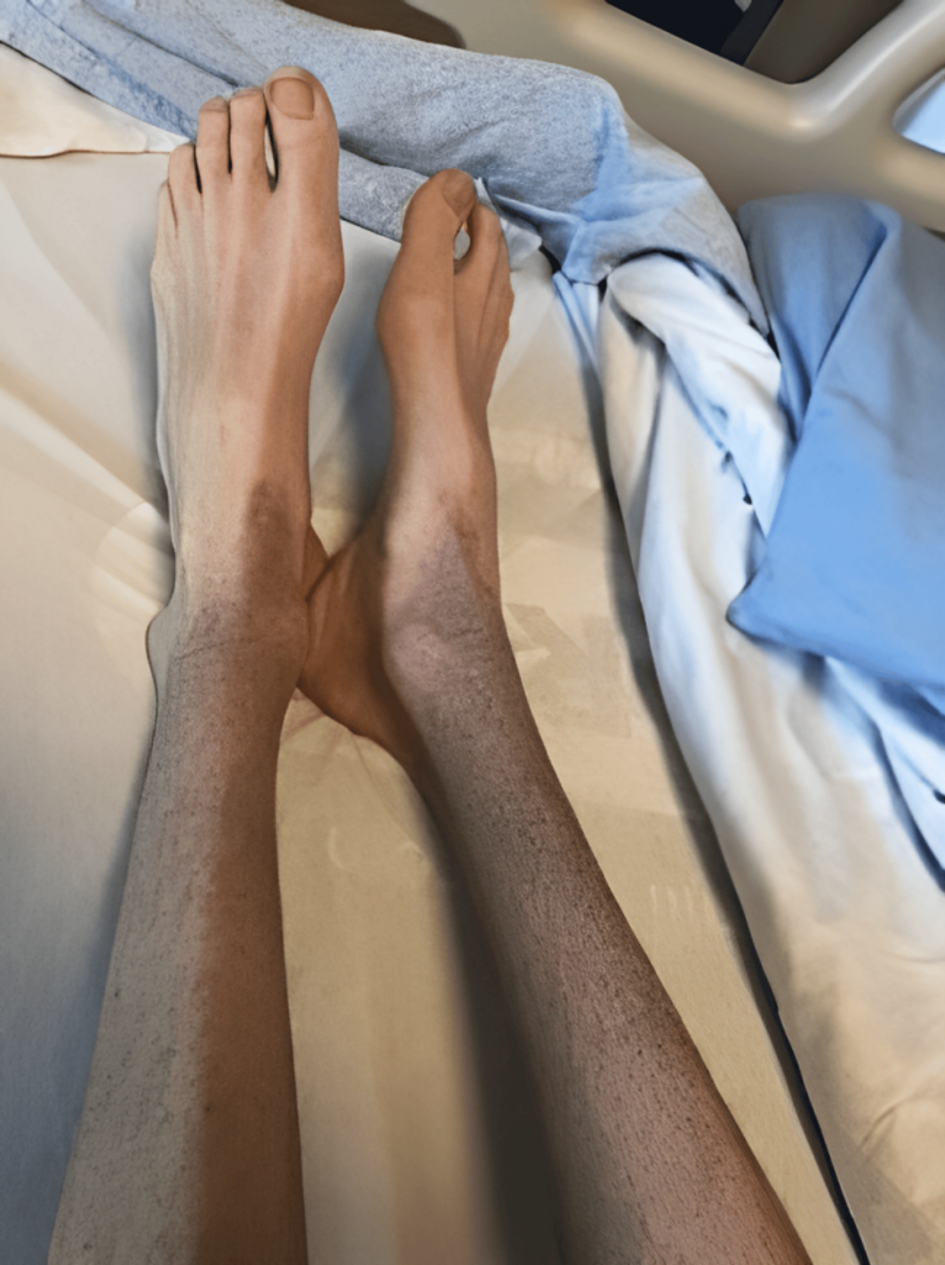
Hyperpigmented perifollicular petechiae and ecchymosis of the shins in a 14-year-old patient with vitamin C deficiency.

Patient anthropometrics were significant for a weight of 30 kg (<0.1 percentile; z-score: <-3), a height of 165.5 cm (87.6 percentile; z-score: 1.1), a body mass index (BMI) of 10.9 (<0.1 percentile, z-score: <-3), and a percent median BMI of 55%. Calculation formula follows: \[ \text{%median BMI} = \left( \frac{\text{Current BMI}}{\text{50th percentile BMI for age and sex}} \right) \times 100 \] It was estimated that the patient lost more than 50% of their premorbid body weight over seven months (with pre-illness weight at 85 percentile weight-for-age and BMI at 83 percentile). Based on these parameters, the patient was classified as having severe malnutrition. The patient’s heart rate was 48 beats per minute and blood pressure was 84/63 mmHg. Physical examination revealed cachexia, cognitive slowing as evidenced by slow responses in conversation and challenges replying to questions, diffuse motor weakness, and acrocyanosis. The dermatological examination was significant for lanugo hair and multiple 1-2 mm hyperpigmented perifollicular petechiae on the extensor surfaces of the forearms, anterior thighs, shins, and lower abdomen, as well as shin ecchymosis (Figure 1). The dental exam was unremarkable.

The patient’s labwork showed mild neutropenia (1.3x10^9/L, reference range: 1.8-8.0x10^9 /L), mild hypokalemia (3.0 mmol/L, reference range: 3.5-5.0 mmol/L), international normalized ratio (INR) of 1.2 (reference range: 0.9-1.2) with partial thromboplastin time (PTT) of 33.8 seconds (reference range: 24-32 seconds). Complete blood count (CBC) was otherwise normal with normal hemoglobin (128 g/L, reference range: 120-160 g/L) and normal MCV. Renal and liver studies were within normal limits. TSH was within normal limits at 1.94 mIU/L (reference range: 0.20-6.50 mIU/L). ECG demonstrated sinus bradycardia, and an echocardiogram demonstrated no abnormal findings.

The patient was admitted to the hospital with a diagnosis of AN-restrictive subtype (AN-R) for management of severe malnutrition, cardiovascular instability, and risk of refeeding syndrome. An evidence-based clinical care guideline for the inpatient management of adolescents with restrictive eating disorders was initiated, which provides recommendations for the safe initiation of nutrition based on the severity of malnutrition at the time of admission to the hospital.

The etiology of this patient’s petechial rash remained unclear in the initial days of admission. While the CBC demonstrated mild neutropenia, an established finding in AN-R, there was no significant thrombocytopenia or evidence of coagulopathy to explain the perifollicular petechial lesions and significant ecchymoses. Given the history of severe malnutrition, a vitamin C deficiency was explored as a potential etiology for the dermatologic findings. Vitamin C is an essential micronutrient, found mainly in fruits and vegetables [8]. In the absence of adequate vitamin C intake over time, clinical manifestations of this nutritional deficiency appear, including those evidenced by the patient.

In this case, the patient had a little-to-daily intake of vitamin C-rich foods for approximately three months. A clinical diagnosis of vitamin C deficiency was made based on the patient’s severe malnutrition, characteristic perifollicular petechial rash, diffuse non-perifollicular petechiae, and ecchymoses. A vitamin C level was not obtained to biochemically confirm the diagnosis, as this patient was diagnosed with scurvy on day four of admission and had been receiving a daily multivitamin containing 90 mg of vitamin C. As the plasma concentration of vitamin C is related to recent intake, a vitamin C level may not have truly reflected the presenting deficiency at the time of diagnosis. The clinical diagnosis was confirmed by the resolution of the rash with effective supplementation of vitamin C. Specifically, treatment was started with a high dose of ascorbic acid, 500 mg by mouth daily, for the duration of one month. The petechial rash began to improve within two weeks, with complete resolution by four weeks of treatment.

## Discussion

To prevent deficiency, the World Health Organization recommends 45 mg/day of vitamin C [8]. Failing to meet the body’s requirements can result in clinical manifestations of scurvy in as little as 8-12 weeks [8]. A study by van Heerden et al. identified children with vitamin C levels below 23 μmol/L as being at risk for scurvy, with symptomatic cases often having even lower levels [9]. Dermatologic findings of this condition include poor wound healing, petechiae, ecchymosis, and hyperkeratosis [2]. Perifollicular hemorrhages are often noted and are localized primarily to the lower extremities, secondary to the effects of gravity-dependent hydrostatic pressure on fragile capillary walls [8]. In the aforementioned clinical case, these characteristic dermatological findings were prominent. Additional mucocutaneous findings seen in scurvy may include gingival hypertrophy and bleeding, epistaxis, corkscrew hairs, and nail findings (koilonychia and splinter hemorrhages) [2]. Musculoskeletal manifestations include painful hemarthrosis, subperiosteal hemorrhages, fractures, and the scorbutic rosary along the costochondral junction [8]. In the current case, an X-ray was not done, though a bone scan was completed later in the admission to evaluate bone mineral density in the context of malnutrition, and this scan did not demonstrate any abnormal findings.

The clinical manifestations of scurvy result from vitamin C’s role in collagen synthesis. In short, vitamin C plays a key role in the hydroxylation and cross-linking of pro-collagen, required for mature collagen synthesis [8]. Mature collagen is an essential component of blood vessel walls and the main constituent of the basement membrane separating the dermis and epidermis of the skin [8]. Vitamin C also participates in various biochemical reactions as a cofactor and antioxidant and plays a role in iron absorption, the body’s inflammatory response, and neurotransmitter biosynthesis [8].

Reports of scurvy in adolescents with AN-R are very rare, likely stemming from the fact that this population typically restricts fat and carbohydrate intake [7]. As a result, literature focusing on scurvy in the eating disorders patient population is limited. Treatment regimens vary, but typical dosing for the treatment of vitamin C deficiency in children ranges from 100 to 300 mg daily up to 1000 mg daily, for periods of about a month or until fully recovered [8]. Interestingly, doses are best given divided, as intestinal and renal mechanisms become oversaturated beyond a 100-mg dose [8]. In this case, treatment was started with a high dose of ascorbic acid (500 mg by mouth daily). The petechial rash began to improve within two weeks, with complete resolution by four weeks of treatment, further supporting the diagnosis.

While scurvy is a rare clinical entity in pediatric clinical practice, reports suggest that this condition is re-emerging in children within specific at-risk populations, including those with autism, eating disorders, inadequate enteral feedings, gastrointestinal malabsorption, end-stage renal disease requiring dialysis, and iron overload from multiple transfusions [8]. Given the treatable nature of this condition, it is important to be vigilant to the possibility of vitamin C deficiency in at-risk patient populations who present with clinical manifestations including petechiae, bruising, and musculoskeletal and dental concerns.

## Conclusions

This case highlights the importance of healthcare provider awareness of at-risk pediatric populations for vitamin C deficiency, including those with physical or neurological disabilities, autism, eating disorders, end-stage renal disease necessitating dialysis, gastrointestinal malabsorption, and inadequate enteral feedings. While rarely seen in clinical practice, this nutritional deficiency is easily treatable and should be considered in the context of a malnourished patient presenting with mucocutaneous, dermatological, and musculoskeletal clinical manifestations. Important dermatological clues include petechiae, perifollicular hemorrhages, ecchymosis, and hyperkeratosis. While not frequently reported in patients with AN, further research is needed to better characterize the incidence of vitamin C deficiency in patients with eating disorders and to inform standardized treatment of this nutritional deficiency.
